# Trends and Outcomes in Lung Transplantation in Patients with and without Idiopathic Pulmonary Fibrosis in Spain during the Period 2016–2020

**DOI:** 10.3390/healthcare11111534

**Published:** 2023-05-24

**Authors:** Belén López-Muñiz Ballesteros, Ana Lopez-de-Andres, Rodrigo Jimenez-Garcia, Jose J. Zamorano-Leon, David Carabantes-Alarcon, Natividad Cuadrado-Corrales, Jose L. Del-Barrio, Napoleon Perez-Farinos, Javier De Miguel-Díez

**Affiliations:** 1 Respiratory Department, Hospital Universitario Infanta Leonor, 28031 Madrid, Spain; 2 Department of Public Health and Maternal & Child Health, Faculty of Medicine, Universidad Complutense de Madrid, 28040 Madrid, Spain; 3 Preventive Medicine and Public Health Teaching and Research Unit, Health Sciences Faculty, Universidad Rey Juan Carlos, 28933 Alcorcón, Spain; 4 Epi-PHAAN Research Group, Instituto de Investigación Biomédica de Málaga (IBIMA), School of Medicine, Universidad de Málaga, 29071 Málaga, Spain; 5 Respiratory Department, Hospital General Universitario Gregorio Marañón, Instituto de Investigación Sanitaria Gregorio Marañón (IiSGM), Faculty of Medicine, Universidad Complutense de Madrid, 28040 Madrid, Spain

**Keywords:** lung transplant, idiopathic pulmonary fibrosis, incidence, complications, in-hospital mortality, COVID-19 pandemic

## Abstract

(1) Background: This paper aims to assess temporal trends (2016–2020) in incidence, patient’s characteristics, complications, length of hospital stay (LOHS) and in-hospital mortality (IHM) among patients with and without idiopathic pulmonary fibrosis (IPF) undergoing lung transplantation (LTx). We also analyse the effect of the COVID-19 pandemic on LTx in these populations. (2) Methods: A retrospective, population-based observational study was conducted using the Spanish National Hospital Discharge Database. Multivariable adjustment was conducted with logistic regression to analyse the IHM. (3) Results: We identified 1777 admissions for LTx during the study period, of which 573 (32.2%) were performed in patients with IPF. The number of hospital admissions for LTx rose from 2016 to 2020, both in patients with and without IPF, but a marked reduction was observed from year 2019 to year 2020. Over time, the proportion of single LTx decreased and bilateral LTx increased significantly in both groups. The incidence of LTx complications increased significantly over time along with the increase in the incidence of IPF. No significant differences in the incidence of complications or in the IHM between patients with and without IPF were found. Suffering any complication of the LTx and pulmonary hypertension were conditions positively associated with IHM in patients with and without IPF. The IHM remained stable from 2016 to 2020 in both study populations and was not affected by the COVID pandemic. (4) Conclusions: Patients with IPF account for almost a third of all lung transplants. The number of LTx increased over time in patients with and without IPF, but a marked reduction was observed from 2019 to 2020. Although the proportion of LTx complications increased significantly over time in both groups, the IHM did not change. IPF was not associated with increased complications or IHM after LTx.

## 1. Introduction

Lung transplantation (LTx) has been shown to improve the expectations and quality of life of patients with advanced lung disease [1]. The International Society for Heart and Lung transplantation has reported an increase in the number of lung transplants in the last 20 years of around 4500 patients since 2017, calculating that 3/4 of these are performed in the United States and Europe (55% and 34%, respectively) [2,3,4]. The diseases in which LTx is performed have changed in recent years, from chronic obstructive pulmonary disease (COPD) being the first indication for LTx in 2007 to diffuse interstitial lung diseases currently, of which 32.4% correspond to patients with idiopathic pulmonary fibrosis (IPF) [5,6]. In this way, up to 60% of lung transplants in the US are due to diffuse interstitial lung diseases [7]. Although in the antifibrotic era there have been improvements in lung function in IPF, it is a progressive disease that will inexorably end in respiratory failure and death after years. That is why the American Thoracic Society, European Respiratory Society, Japanese Respiratory Society, and Latin American Thoracic Society societies make a strong recommendation in favour of LTx in these patients, which represents a 75% increase in survival compared to pharmacological treatment, having also proven to be the only way to improve symptoms, quality of life, and survival over time [8,9,10]. Despite the fact that the indications for LTx in patients with IPF are changing, it is important to identify which patients could benefit from this treatment, as well as the time and age of the indication, and pay attention to comorbidities such as gastroesophageal reflux, pulmonary hypertension, obesity, or diabetes mellitus, as they may increase the risk of transplant complications [11,12,13,14].

LTx can be single or double. In recent years, an increase in the number of double-lung transplants has been detected in the United States and Europe. Although single-LTx means less time on the surgical waiting list, shorter procedure time, and lower risk of complications, double-LTx has shown greater survival and fewer chronic graft-versus-host rejections. Median post-transplant survival is 50% at 5 years [15,16,17].

The objectives of our study were as follows: (a) to assess the temporal trends in the incidence of LTx in Spain from 2016 to 2020 among subjects suffering or not from IPF; (b) to describe and compare the clinical conditions, such as comorbidities, complications of the transplanted lung, and post-transplant infections of these two populations; (c) to analyse the temporal trends in the length of hospital stay (LOHS) and the in-hospital mortality (IHM) among IPF and non-IPF patients who received a LTx; and (d) to assess whether the COVID 19 pandemic has affected the number or the outcomes of LTx in these patients.

## 2. Materials and Methods

We conducted an observational study with data obtained from the Registro de Actividad de Atención Especializada-Conjunto Mínimo Básico de Datos (RAE-CMBD). This is a hospital discharge database that collects information on all admissions to public and private hospitals in Spain [18]. We analysed all registries included in the RAE-CMBD from 1 January 2016 to 31 December 2020.

The variables collected by this registry include sex, data of birth, dates of admission and discharge, and if the patient has died in the hospital. Additionally, for each patient, and using the International Classification of Disease 10th version (ICD-10), up to 20 diagnoses and procedures can be recorded. 

The study population included subjects who had a code for LTx (ICD-10 codes OBYxxxx, in any procedure field); LTx were classified as single or bilateral LTx using the ICD-10 codes shown in Supplementary Table S1. Patients with heart and LTx were excluded.

We grouped admissions by IPF status. We considered IPF patients as those with any ICD10 codes J84.1xx in any diagnostic position, and the rest as non-IPF patients.

To assess the presence of comorbidities, we used the Charlson comorbidity index (CCI), using ICD10 codes described by Sundararajan et al. [19]. The CCI was analysed as a sum for each patient, categorized in none and one or more conditions and providing results for each of the specific conditions included in the index. Likewise, regardless of the diagnostic position, the presence of COVID-19 in year 2020 and the presence of pulmonary hypertension, COPD, asthma, and current tobacco use were evaluated (Table S1).

Procedures such as haemodialysis, extracorporeal membrane oxygenation, and tracheostomy were identified in any of the procedures fields of the database (Table S1).

The following complications of LTx were considered if recorded in any diagnostic position: lung transplant rejection, lung transplant failure, lung transplant infection, and other or unspecified complications of lung transplant (Table S1). Complications were analysed individually, and if one or more appeared they were designated with the variable “any complication of LTx”. 

In addition, we evaluated the presence of pneumonia and ventilator-associated pneumonia as complications (Table S1).

*Staphylococcus* bacteraemia, Gram-negative bacteraemia, *Pseudomonas aeruginosa* infection, *Aspergillus* infection, and Cytomegalovirus infection were identified in the database using the corresponding ICD10 diagnosis codes (Table S1).

### 2.1. Statistical Analysis

We performed a descriptive analysis calculating mean with standard deviation (SD) for continuous variables and total frequencies with proportions for categorical variables. 

To assess changes over time we used Cochran–Armitage tests for categorical variables, and the test for linear trend or the Jonckheere–Terpstra test for continuous variables. 

We compared categorical variables using the χ^2^ test and continuous variables with the *t*-test or the Mann–Whitney test, as required.

We conducted three multivariable logistic regression models (IPF population, non-IPF population, and the entire population) with IHM as the dependent variable. 

To construct the multivariable models, the steps were as follows: 1. Bivariate analysis to assess the association of independent variables with the IHM. 2. We selected for the multivariable analysis those variables with a significant association in the bivariate tests, as well as other study variables that have been found relevant in previous investigations. 3. The Wald statistic was used to decide, one by one, if variables were included in the model. Using the likelihood ratio test, consecutive models were compared with the previous ones as new variables were added. 4. For the final model, linearity and interactions between variables in the model was checked to identify which study variables were independently associated with IHM. 

Odds ratios (ORs) are provided as measure of association along with their 95% confidence interval (95%CI).

All statistical analysis was performed with Stata version 14 (Stata, College Station, TX, USA). A *p* value < 0.05 (2-sided) was considered significant.

### 2.2. Ethics Statement

The anonymized database of the RAE-CMBD was provided to us by the Spanish Ministry of Health upon request, and after scientifical and ethical evaluation of our study protocol [20]. In Spain, the law allows the inclusion of patient’s information in administrative databases without written consent.

## 3. Results

From 2016 to 2020, we identified 1777 admissions for LTx in Spain. Over the study period, 573 (32.2%) of all admissions for LTx were performed in IPF-patients. Of these, 291 were single (50.7%) and the remaining bilateral LTx. 

**Figure 1:**
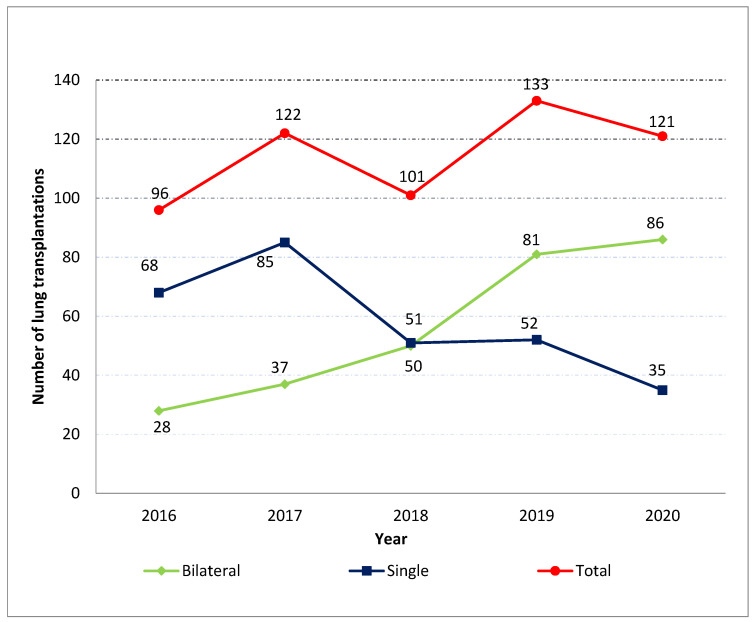
Number of lung transplantations among patients with idiopathic pulmonary fibrosis in Spain from 2016 to 2020 according to single or bilateral procedure.

The number hospital admissions for LTx (single and bilateral) in IPF-patients can be seen in Figure 1. The total number increased 26% from 2016 to 2020. The number of single LTx decreased by 48.5% between 2016 and 2020 from 68 to 35, whereas bilateral increased by 207%. 

Table 1 shows the sociodemographic and clinical conditions of patients who underwent LTx, according to the presence of IPF. The number of bilateral LTx patients increased (117 in 2016 vs. 176 in 2020) among non-IPF patient, and the number of single LTx decreased from 98 to 43.

The proportion of single LTx decreased significantly over time in patients with and without IPF (70.8% and 45.6% in 2016 vs. 28.9% and 19.6% in 2020; all *p* < 0.001); however, bilateral LTx increased significantly in both groups (in IPF group: 29.2% in 2016 vs. 71.1% in 2020; in non-IPF group; 54.4% in 2016 vs. 80.4% in 2020; all *p* < 0.001). Over the entire study period, IPF patients underwent more single LTx than non-IPF patients (50.8% vs. 28.9%; *p* < 0.001); however, they underwent less bilateral LTx (49.2% vs. 71.1%; *p* < 0.001).

A marked reduction was observed from year 2019 to year 2020 among both groups of patients in the number of LTx (in IPF group: 52 in 2019 vs. 35 in 2020; and in non-IPF group: 50 in 2019 vs. 43 in 2020). 

Among the patients who underwent LTx, there were fewer women than men and proportionally fewer women in the IPF group vs. the non-IPF group (24.6% vs. 40.9%; *p* < 0.001). 

The age ranged from 0 to 72 years for those without IPF and from 13 to 71 years for those with this condition. Over the entire study period, the mean age of IPF patients who underwent LTx was almost 7 years higher than that of those patients without IPF (58.1; SD 9.0 vs. 51.4; SD 14.2, *p* < 0.001). Only in non-IPF patients was mean age increased significantly (*p* = 0.025) between 2016 and 2020.

According to the mean CCI, patients without IPF had more comorbidities than those with IPF (*p* < 0·001). 

The total mean LOHS was significantly shorter in the IPF patients (44.7 days) than the non-IPF patients (48.7 days, *p* < 0.001). However, the IHM was similar, 11.5% for IPF and 11.9% for non-IPF patients. Neither LOHS nor IHM changed significantly from 2016 to 2020 in any study group.

Table 2 shows the prevalence of comorbidities and procedures on hospitalized patients who underwent LTx, according to the presence of IPF.

Regarding comorbidities, in patients with IPF, the prevalence of pulmonary hypertension increased over time (21.9% in 2016 vs. 33.0% in 2020; *p* = 0.013). In the non-IPF group, a significantly increase in the frequency of moderate/severe liver disease and hemiplegia/paraplegia between 2016 and 2020 was observed. However, the prevalence of renal disease and cancer/metastatic solid tumour decreased overtime. The prevalence of rheumatoid disease was significantly higher in the IPF group than in the non-IPF group (7.9% vs. 3.5%; *p* < 0.001). Patients without IPF had a significantly higher prevalence of COPD (56.1% vs. 13.1%) and asthma (2.3% vs. 0.9%) than those with IPF. Current tobacco use was not codified in any IPF patient and only three non-IPF. 

As can been seen in Table 2, in the IPF and non-IPF groups the use of extracorporeal membrane oxygenation increased significantly between 2016 and 2020 (IPF group: 14.6% vs. 24.8%, *p* = 0.036 and in non-IPF group: 14.4% vs. 26.9%, *p* < 0.001).

Table 3 shows the complications and pathogens isolations after LTx among patients with and without IPF.

Around 25% of the patients undergoing LTx suffered LTx rejections, and another 11% LTx infection. If any possible complications were considered, 51.0% and 49.8% of patients with and without IPF were affected. None of the LTx complications showed significant differences when patients with and without IPF were compared.

The proportion of LTx complications showed a significant increase over time in both groups analysed, specifically lung transplant rejection, lung transplant failure, other/unspecified complications of lung transplant, and any complication of lung transplant. Furthermore, the proportion of lung transplant infection in the IPF group increased significantly, almost four times, between 2016 and 2020 (3.1% vs. 12.4%; *p* = 0.004).

Pneumonia and ventilator-associated pneumonia during the hospital admission were diagnosed in 6.1% and 1.4% of patients with IPF and 8.1% and 1.3% among non-IPF patients (Table 3). 

Regarding isolated pathogens, those most frequently found among IPF patients were *Staphylococcus* bacteraemia (11.7%), followed by *Pseudomonas aeruginosa* (8.5%), and Gram-negative bacteraemia (6.6%). A very similar prevalence was codified among non-IPF patients. 

Shown in Table S2 is the IHM in patients with IPF who underwent a LTx according to study variables. 

IHM was higher in patients with IPF presenting with pulmonary hypertension than those without this condition (20.9% vs. 9.1%; *p* < 0.001). Crude IHM in patients with IPF was higher in single LTx, in women, in patients with higher CCI, and in those with any complications of lung transplant; however, all these differences were not significant.

The results of comparing the characteristics and the IHM of patients with IPF who underwent a LTx in years 2019 and 2020 can be seen in Table S3. 

Patients operated on in year 2020 had pulmonary hypertension coded in a higher proportion that those who underwent LTx in year 2019 (33.0% vs. 14.2%; *p* < 0.001). IHM in patients with IPF was slightly higher in 2020 (14.0% in 2020 and 12.0% in 2019), but this difference was not significant. No differences were found between 2020 and 2019 in the IHM after stratification by type of procedure, sex, age, CCI, pulmonary hypertension, and any complication of lung transplant.

The results of the multivariable analysis to identify factors associated with IHM after LTx in Spain from 2016–2020 in patients with and without IPF is shown in Table 4. 

Among patients with IPF, suffering any complication of the lung transplant (OR 1.47; 95%CI 1.01–2.55) and pulmonary hypertension (OR 2.48; 95%CI 1.43–4.31) were conditions positively associated with IHM. These two same variables showed a significant association among those without IPF and in the entire population of LTx patients (Table 4). As found in the bivariate analysis, the IHM did not changed significantly from 2016 to 2020 in any study population.

Finally, from 2016 to 2020, and after adjusting by all study variables, IPF was not associated with IHM after LTx (OR 1.13; 95%CI 0.8–1.59).

## 4. Discussion

In our study, we identified 1777 patients who received LTx in Spain during the period 2016–2020. Of them, 32.5% were patients with IPF, evidencing an increase in transplants in patients with this disease over time. However, in 2020 there was a marked reduction, both in the group of patients with IPF and in those without IPF. This reduction was due to the SARS-CoV-2 pandemic, and it has also been detected in other series. Thus, in England, the average number of transplants in the first months of the pandemic was 4 per month, while in the months of January and February 2020 it was 12.5 per month [21]. In addition, the number of deceased donors from whom the lungs were offered for transplantation fell by 48% in this series, mirroring the published experience of other lung transplant programs, globally [22,23]. Although the antifibrotic drugs Pirfenidone et al. [24] and Nintedanib et al. [25] have shown less decline in lung function and improved survival, there continues to be an inexorable progression of the disease towards the development of respiratory failure, pulmonary hypertension, and death, so in advanced stages of the disease, the only treatment that has been shown to improve quality of life and survival is LTx [9,13]. In fact, there is currently an increase in the number of patients with IPF transplanted, as has been reported by the International Society for Heart and Lung transplantation [3]. In Spain, IPF is the main indication for LTx. Thus, according to data from the National Transplant Organization (ONT), in 2017 they accounted for 36% of the patients included on the waiting list and 39% of the recipients had IPF [26].

LTx can be single or double, with the advantages of the former being shorter surgical time, less trauma, shorter ischemia time, and shorter waiting time on the surgical list [27]. The evidence for performing one or the other procedure is poor, despite the large number of comparative studies that have been described [28,29,30,31]. In our series, single-LTx continues to be more frequent in the group of patients with IPF. However, in both groups a tendency to perform a greater number of double-lung transplants over time was detected, with no significant differences in mortality or complications. In the same way, a recent meta-analysis showed that double-LTx was associated with better postoperative function, but no differences were found in long-term survival between the two groups [32]. On the other hand, in the series by Spratt et al., who analysed 151 patients with IPF who underwent transplants between 2005 and 2017, no significant differences were detected between single or double lung transplants [33]. Although the recommendations to date are not well established and should be individualized for each patient, these authors proposed reserving single-LTx for older patients with pulmonary hypertension due to shorter waiting time on the list and shorter surgical time, thus reducing possible complications in more severe patients [26].

In advanced stages, lung diseases are associated with many comorbidities, both respiratory and non-respiratory, which affect the quality of life of patients and may interfere with the transplant prognosis [14,34]. In our series, the group of patients with IPF were mainly male and younger than the group without IPF, and we detected a greater number of comorbidities over time in both groups, with pulmonary hypertension being the most frequent in the group with IPF. The presence of pulmonary hypertension in patients with IPF is highly variable, ranging from 3% to 86% [14]. In our series, it was found in 23.4% of the patients with IPF and in 21.3% of those without IPF. Due to the high frequency of pulmonary hypertension, it is important to perform a correct assessment of pulmonary artery pressure and right ventricular function in all those patients who are going to undergo a lung transplant, since the increase in pulmonary artery pressure is associated with increased graft dysfunction and postoperative mortality [35]. On the other hand, the association of IPF and pulmonary hypertension in our series led to an increase in mortality compared to the group without pulmonary hypertension (20.9% vs. 9.1%). This is since pulmonary hypertension is one of the main causes of peri-surgical mortality and with the lowest survival at one year. Despite these facts, in recent years, improvements in the surgical technique, the use of extracorporeal membrane oxygenation, and better management of acute rejection have contributed to improve survival in these patients [36,37].

As in our series, the use of extracorporeal membrane oxygenation is becoming widespread. However, the results obtained in the different series are contradictory and inconclusive. Thus, there are authors who have associated its use with a higher rate of complications [38], while others have reported a lower rejection rate in those patients treated with extracorporeal membrane oxygenation during surgery [39]. In any case, the development of acute rejection could be related to the use of blood products during surgery, the fraction of oxygen required, and the clamping technique [40,41].

Another complication in our series, which has been multiplied by four from 2016 to 2020, are infections (3.1% vs. 12.4%, respectively). More specifically, pneumonia occurred in 6.1% of patients with IPF in 2016, compared to 8.1% in 2020. Infections are one of the main post-transplant complications, having been described as the second cause of death. Of these, the most common are respiratory infections, mostly bacterial. In the Spanish series by Aguilar et al., bacterial pneumonia 4 weeks after transplantation occurred in up to 44% of transplanted patients [42]. In our series, as in others studied [40,43], the most frequent pathogen found in solid organ transplants was Staphylococcus aureus. Infections by multi-resistant microorganisms are usually less frequent and are related to previous colonization, both by the donor and the recipient [44,45]. In this way, Bunsow et al. analysed the prevalence of multi-resistant bacteria in 268 lungs from donors to assess their transmission to the recipient, noting that out of 56.8% detected in the donor, only 4.9% developed in the recipient. Additionally, of these, only one patient had pneumonia [46]. On the other hand, cytomegalovirus infections have decreased in frequency to date, possibly because of specific prophylaxis [47].

Overall, it has been previously described that the survival of transplanted patients is 89% at 3 months, 80% at one year, and 31% at 10 years. The highest number of reported cases of mortality occur in the first 30 days, due to high rejection or infections, mainly by cytomegalovirus [48,49]. The group of patients with diffuse interstitial lung diseases are those with the worst survival rate and the highest IHM, ranging from 10% to 20% [40]. 

In our series, we did not observe an increase in IHM over time in either of the two groups. The overall IHM of patients with IPF was 12.7% in patients with a single-lung transplant versus 11.0% in those with a double-lung transplant. These mortality results are similar to those described in the Spanish series by Prudencio-Ribera et al., who detected a figure of 13.4% in the group of patients with diffuse interstitial lung disease, with IPF being the most frequent disease, detected in 79.6% of patients [48]. It is worth noting the increase in IHM in 2020 in both groups of patients (not significant), possibly due to the SARS-CoV-2 pandemic, as has been described in other series [21]. On the other hand, IHM in our series was higher in patients with a single-lung transplant, in women, and in those with greater comorbidity, although the results were not statistically significant. The International Society for Heart and Lung transplantation shows that the main risk factors for short-term mortality are single-LTx, the IPF itself, and being male [50]. In the series by Urlik et al., IHM was higher in patients who received a single-lung transplant and in those in whom the donor was older [51]. On the other hand, in the study by Mosher et al., the older patients with more comorbidities had the higher IHM [52].

Early IHM in single-LTx has shown contradictory results, with an increase in the series by Arango et al. [49] and Force et al. [16], who recommend resorting to double-LTx in younger patients. On the other hand, in the series by Prudencio-Ribera et al., although long-term survival was higher in the double-LTx group, no significant differences were found at one month between the two options [48]. In any case, the main causes of IHM are post-transplant complications. In our series, 50.9% of the patients with IPF and 49.8% of the patients without IPF had some complication. On the other hand, 25% of the patients with IPF suffered acute rejection of the graft and 11% infections. We observed an increase over time in the rate of complications, mainly acute rejection. Precisely, in the literature, it has been described that this is the main cause of early post-transplant morbidity and mortality. To avoid it, it is important to make a correct selection of the donor and the recipient [53]. 

Our study has several limitations. The main one is that the data have been obtained from an administrative database, in which the pathologies and procedures are coded according to the ICD-10-CM. The validity of the RAE-CMBD in identifying LTx has not been assessed; however, studies conducted in the US, using the National Inpatient Sample, concluded that discharge databases are reliable and accurate for national epidemiological investigations [54]. Furthermore, our global results are very similar to those reported by the Spanish National Registry of Lung Transplantation [55]. Secondly, when collecting the data from an administrative database, there may be some incomplete cases, depending on the centre that entered the information. Third, only one post-transplant analysis was performed, not considering the patients who developed complications or died on the waiting list or after hospital discharge. Fourth, as it is a heterogeneous population group, with different pathologies, the level of severity prior to admission was not collected. Fifth, due to the study design, we cannot establish the causality in the associations found. Sixth, the lung allocation score has not been considered, which may influence the selection of candidate patients for transplantation and its success [56]. Finally, we unfortunately do not have data about the pandemic up to the year 2021; this would be relevant because vaccination coverage was still not optimal and there are no data about the Beta 1.1 SARS-CoV-2 variant, which was very aggressive.

Future works, including more detailed clinical and biological data and information on long term mortality after LTx, should be conducted in our country to improve the knowledge and management of patients with IPF.

## 5. Conclusions

In conclusion, patients with IPF account for almost a third of all lung transplants. The number of LTx increased over time in patients with and without IPF, but a marked reduction was observed from 2019 to 2020. The proportion of single LTx decreased significantly, while bilateral LTx increased significantly among both groups. Although the proportion of LTx complications increased significantly over time, the IHM did not change. IPF was not associated with IHM after LTx.

## Figures and Tables

**Table 1:** Sociodemographic and clinical conditions of patients that underwent a lung transplantation in Spain from 2016 to 2020 according to the presence of a diagnosis of idiopathic pulmonary fibrosis (IPF).

		2016		2017		2018		2019		2020		TOTAL	
		No IPF	IPF	No IPF	IPF	No IPF	IPF	No IPF	IPF	No IPF	IPF	No IPF	IPF
N. of transplantations		215	96	228	122	260	101	282	133	219	121	1204	573
Type, n (%)	Single ^abc^	98 (45.6)	68 (70.8)	104 (45.6)	85 (69. 7)	53 (20.4)	51 (50.5)	50 (17.7)	52 (39.1)	43 (19.6)	35 (28.9)	348 (28.9)	291 (50.8)
	Bilateral ^abc^	117 (54.4)	28 (29.2)	124 (54.4)	37 (30.3)	207 (79.6)	50 (49.5)	232 (82.3)	81 (60.9)	176 (80.4)	86 (71.1)	856 (71.1)	282 (49.2)
Female sex, n (%) ^c^		83 (38.6)	25 (26.0)	88 (38.6)	32 (26.2)	114 (43.9)	26 (25.7)	116 (41.1)	37 (27.8)	91 (41.6)	21 (17.4)	492 (40.9)	141 (24.6)
Age, mean (SD) ^bc^		49.0 (15.9)	56.4 (9.5)	50.5 (13.4)	58.1 (8.9)	51.6 (13.6)	58.4 (8.5)	52.7 (13.7)	58.2 (9.2)	52.6 (14.3)	59.1 (9.0)	51.4 (14.2)	58.1 (9.0)
<45 years, n (%) ^c^		63 (29.3)	11 (11.5)	63 (27.6)	11 (9.0)	61 (23.5)	6 (6.0)	63 (22.3)	12 (9.0)	49 (22.4)	5 (4.1)	299 (24.8)	45 (7.9)
45–54 years, n (%) ^c^		42 (19.5)	14 (14.6)	45 (19.7)	20 (16.4)	48 (18.5)	16 (15.8)	50 (17.7)	21 (15.8)	36 (16.4)	17 (14.0)	221 (18.4)	88 (15.4)
55–64 years, n (%) ^c^		91 (42.3)	52 (54.2)	105 (46.1)	63 (51.6)	130 (50.0)	60 (59.4)	130 (46.1)	72 (54.1)	110 (50.2)	72 (59.5)	566 (47.0)	319 (55.7)
≥65 years, n (%) ^c^		19 (8.8)	19 (19.8)	15 (6.6)	28 (23.0)	21 (8.1)	19 (18.8)	39 (13.8)	28 (21.1)	24 (11.0)	27 (22.3)	118 (9.8)	121 (21.1)
CCI, n (%)	0 ^c^	38 (17.7)	53 (55.2)	28 (12.3)	55 (45.1)	56 (21.5)	48 (47.5)	49 (17.4)	61 (45.9)	36 (16.4)	57 (47.1)	207 (17.2)	274 (47.8)
	≥1 ^c^	177 (82.3)	43 (44.8)	200 (87.7)	67 (54.9)	204 (78.5)	53 (52.5)	233 (82.6)	72 (54.1)	183 (83.6)	64 (52.9)	997 (82.8)	299(52.2)
CCI, mean (SD) ^c^		1.2 (0.9)	0.78 (1.2)	1.25 (0.9)	0.78 (0.9)	1.09 (0.9)	0.76 (0.9)	1.29 (1.1)	0.81 (1.0)	1.26 (1.0)	0.86 (1.2)	1.22 (1.0)	0.8 (1.0)
LOHS, mean (SD) ^c^		54.2 (53.8)	48.5 (42.4)	46.8 (37.5)	42.1 (32.3)	47.6 (42.3)	44.8 (39.4)	49.0 (37.4)	49.6 (40.9)	46.3 (41.9)	39.1 (26.1)	48.7 (42.6)	44.7 (36.5)
IHM, n (%)		21 (9.8)	13 (13.5)	22 (9.7)	15 (12.3)	32 (12.3)	7 (6.9)	34 (12.1)	16 (12.0)	29 (13.2)	17 (14.1)	138 (11.5)	68 (11.9)

CCI: Charlson comorbidity index; LOHS: length of hospital stay; IHM: in-hospital mortality. ^a^ *p* value for time trend for among IPF patients < 0.05. ^b^ *p* value for time trend for among non-IPF patients < 0.05. ^c^ *p* value for comparison of total values between patients with and without IPF.

**Table 2:** Comorbidities included in the Charlson comorbidity index, pulmonary hypertension and procedures among hospitalized patients with and without idiopathic pulmonary fibrosis (IPF) who underwent a lung transplantation in Spain from 2016 to 2020.

	IPF	2016	2017	2018	2019	2020	TOTAL
Myocardial infarction, n (%)	Yes	0 (0)	2 (1.6)	2 (2.0)	1 (1.0)	1 (0.8)	6 (1.1)
	No	2 (0.9)	2 (0.9)	2 (0.8)	9 (3.2)	3 (1.4)	18 (1.5)
Congestive heart failure, n (%)	Yes	5 (5.2)	11 (9.0)	9 (9.0)	7 (5.3)	7 (5.8)	39 (6.8)
	No	11 (5.1)	13 (5.7)	9 (3.5)	21 (7.5)	7 (3.2)	61 (5.1)
Peripheral vascular disease, n (%)	Yes	0 (0)	1 (0.8)	2 (2.0)	1 (0.8)	5 (4.1)	9 (1.6)
	No	4 (1.9)	3 (1.3)	5 (1.9)	6 (2.1)	6 (2.7)	24 (2.0)
Cerebrovascular disease, n (%)	Yes	3 (3.1)	3 (2.5)	2 (2.0)	4 (3.0)	6 (5.0)	18 (3.1)
	No	7 (3.3)	7 (3.1)	10 (3.9)	4 (1.4)	14 (6.4)	42 (3.5)
Dementia, n (%)	Yes	0 (0)	0 (0)	0 (0)	1 (0.8)	0 (0)	1 (0.2)
	No	0 (0)	0 (0)	0 (0)	0 (0)	0 (0)	0 (0)
Rheumatoid disease, n (%) ^b^	Yes	9 (9.4)	12 (9.8)	7 (6.9)	10 (7.5)	7 (5.8)	45 (7.9)
	No	6 (2.8)	11 (4.8)	11 (4.2)	8 (2.8)	6 (2.7)	42 (3.5)
Peptic ulcer disease, n (%)	Yes	1 (1.0)	0 (0)	0 (0)	0 (0)	2 (1.7)	3 (0.5)
	No	1 (0.5)	2 (0.9)	3 (1.2)	2 (0.7)	2 (0.9)	10 (0.8)
Moderate or severe liver disease, n (%)	Yes	3 (3.1)	5 (4.1)	8 (7.9)	12 (9.0)	8 (6.6)	36 (6.3)
	No ^a^	9 (4.2)	13 (5.7)	14 (5.4)	32 (11.4)	22 (10.1)	90 (7.5)
Diabetes and diabetes with complications, n (%)	Yes	10 (10.4)	19 (15.6)	15 (14.9)	19 (14.3)	10 (8.3)	73 (12.7)
	No	29 (13.5)	25 (11.0)	28 (10.8)	23 (8.2)	17 (7.8)	122 (10.1)
Hemiplegia or paraplegia, n (%)	Yes	0 (0)	2 (1.6)	0 (0)	0 (0)	0 (0)	2 (0.4)
	No ^a^	3 (1.4)	0 (0)	0 (0)	2 (0.7)	5 (2.3)	10 (0.8)
Renal disease, n (%)	Yes	1 (1.0)	1 (0.8)	1 (1.0)	3 (2.3)	3 (2.5)	9 (1.6)
	No ^a^	6 (2.8)	12 (5.3)	4 (1.5)	3 (1.1)	5 (2.3)	30 (2.5)
Cancer and metastatic solid tumour, n (%)	Yes	2 (2.1)	1 (0.8)	1 (1.0)	2 (1.5)	7 (5.8)	13 (2.3)
	No ^a^	6 (2.8)	3 (1.3)	5 (1.9)	15 (5.3)	4 (1.8)	33 (2.7)
AIDS/HIV, n (%)	Yes	0 (0)	0 (0)	0 (0)	0 (0)	0 (0)	0 (0)
	No	0 (0)	0 (0)	1 (0.38)	0 (0)	1 (0.5)	2 (0.2)
Pulmonary hypertension, n (%)	Yes ^a^	21 (21.9)	29 (23.8)	25 (24.8)	19 (14.3)	40 (33.1)	134 (23.4)
	No	47 (21.9)	49 (21.5)	57 (21.9)	53 (18.8)	50 (22.8)	256 (21.3)
Chronic Obstructive Pulmonary Disease, n (%) ^b^	Yes	12 (12.5)	12 (9.8)	16 (15.8)	20 (15.0)	15 (12.4)	75 (13.1)
	No	113 (52.6)	120 (52.6)	140 (53.9)	167 (59.2)	135 (61.6)	675 (56.1)
Asthma, n (%) ^b^	Yes	2 (2.1)	1 (0.8)	0 (0)	2 (1.5)	0 (0)	5 (0.9)
	No	3 (1.4)	4 (1.8)	8 (3.1)	9 (3.2)	4 (1.8)	28 (2.3)
Current tobacco use, n (%)	Yes	0 (0)	0 (0)	0 (0)	0 (0)	0 (0)	0 (0)
	No	1 (0.5)	0 (0)	2 (0.8)	0 (0)	0 (0)	3 (0.3)
Haemodialysis, n (%)	Yes	3 (3.1)	5 (4.1)	4 (4.0)	11 (8.3)	12 (9.9)	35 (6.1)
	No	18 (8.4)	22 (9.7)	18 (6.9)	18 (6.4)	19 (8.7)	95 (7.9)
Extracorporeal membrane oxygenation, n (%)	Yes ^a^	14 (14.6)	14 (11.5)	22 (21.8)	31 (23.3)	30 (24.8)	111 (19.4)
	No ^a^	31 (14.4)	39 (17.1)	45 (17.3)	76 (27.0)	59 (26.9)	250 (20.8)
Tracheostomy, n (%)	Yes	23 (24.0)	24 (19.7)	24 (23.8)	39 (29.3)	27 (22.3)	137 (23.9)
	No	45 (20.9)	49 (21.5)	68 (26.2)	63 (22.3)	45 (20.6)	270 (22.4)

^a^ *p* value for time trend < 0.05; ^b^ *p* value for comparison of total values between patients with and without IPF.

**Table 3:** Lung transplant complications, pneumonia and pathogen isolations among hospitalized patients with and without idiopathic pulmonary fibrosis (IPF) who underwent a lung transplantation in Spain from 2016 to 2020.

	IPF	2016	2017	2018	2019	2020	TOTAL
Lung transplant rejection, n (%)	Yes ^a^	15 (15.6)	20 (16.4)	32 (31.7)	39 (29.3)	36 (29.8)	142 (24.8)
	No ^a^	35 (16.3)	29 (12.7)	84 (32.3)	102 (36.2)	56 (25.6)	306 (25.4)
Lung transplant failure, n (%)	Yes ^a^	1 (1.1)	7 (5.7)	12 (11.9)	17 (12.8)	16 (13.2)	53 (9.3)
	No ^a^	8 (3.7)	10 (4.4)	28 (10.8)	22 (7.8)	16 (7.3)	84 (7.0)
Lung transplant infection, n (%)	Yes ^a^	3 (3.1)	10 (8.2)	15 (14.9)	25 (18.8)	15 (12.4)	68 (11.9)
	No	18 (8.4)	22 (9.7)	39 (15.0)	35 (12.4)	29 (13.2)	143 (11.9)
Other or unspecified complications of lung transplant, n (%)	Yes ^a^	19 (19.8)	32 (26.2)	21 (20.8)	15 (11.3)	17 (14.1)	104 (18.2)
	No ^a^	49 (22.8)	61 (26.8)	43 (16.5)	43 (15.3)	23 (10.5)	219 (18.2)
Any complication of lung transplant, n (%)	Yes ^a^	36 (37.5)	58 (47.5)	64 (63.4)	71 (53.4)	63 (52.1)	292 (51.0)
	No ^a^	89 (41.4)	104 (45.6)	151 (58.1)	158 (56.1)	98 (44.8)	600 (49.8)
Pneumonia, n (%)	Yes	6 (6.3)	5 (4.1)	4 (4.0)	10 (7.5)	10 (8.3)	35 (6.1)
	No	18 (8.4)	18 (7.9)	26 (10.0)	18 (6.4)	18 (8.2)	98 (8.1)
Ventilator-associated Pneumonia, n (%)	Yes	1 (1.1)	2 (1.6)	1 (1.0)	3 (2.3)	1 (0.8)	8 (1.4)
	No	2 (0.9)	2 (0.9)	2 (0.8)	3 (1.1)	7 (3.2)	16 (1.3)
*Staphylococcus* bacteraemia, n (%)	Yes	9 (9.4)	12 (9.8)	11 (10.9)	20 (15.1)	15 (12.4)	67 (11.7)
	No	23 (10.7)	26 (11.4)	33 (12.7)	27 (9.6)	26 (11.9)	135 (11.2)
Gram-negative bacteraemia, n (%)	Yes	7 (7.3)	9 (7.4)	6 (5.9)	8 (6.1)	8 (6.6)	38 (6.6)
	No	15 (7.0)	12 (5.3)	17 (6.5)	23 (8.2)	15 (6.9)	82 (6.8)
*Pseudomonas aeruginosa*, n (%)	Yes	5 (5.2)	11 (9.1)	13 (12.9)	11 (8.3)	9 (7.4)	49 (8.5)
	No	20 (9.3)	16 (7.1)	32 (12.3)	19 (6.7)	23 (10.5)	110 (9.1)
*Aspergillus*, n (%)	Yes	6 (6.3)	2 (1.6)	6 (5.9)	6 (4.5)	3 (2.5)	23 (4.1)
	No	11 (5.1)	7 (3.1)	15 (5.8)	23 (8.2)	10 (4.6)	66 (5.5)
Cytomegalovirus, n (%)	Yes	2 (2.1)	3 (2.5)	5 (5.0)	10 (7.5)	2 (1.7)	22 (3.8)
	No	3 (1.4)	10 (4.4)	19 (7.3)	13 (4.6)	17 (7.8)	62 (5.2)

^a^ *p* value for time trend < 0.05.

**Table 4:** Multivariable analysis of the factors associated with mortality during hospital admission for lung transplantation in Spain, 2016–2020 according to the presence of idiopathic pulmonary fibrosis (IPF).

		IPF	NO IPF	BOTH
		OR (95%CI)	OR (95%CI)	OR (95%CI)
Sex	Men	1	1	1
	Women	1.34 (0.75–2.39)	1.14 (0.79–1.65)	1.2 (0.88–1.63)
Age groups	<45 years	1	1	1
	45–54 years	1.15 (0.41–3.23)	0.9 (0.52–1.55)	0.92 (0.58–1.48)
	55–64 years	0.94 (0.37–2.36)	0.9 (0.57–1.43)	0.88 (0.59–1.31)
	≥65 years	0.55 (0.18–1.65)	0.83 (0.41–1.7)	0.64 (0.36–1.13)
CCI index	0	1	1	1
	≥1	1.33 (0.78–2.28)	0.83 (0.51–1.34)	1.07 (0.75–1.53)
Any complication of lung transplant	Yes	1.47 (1.01–2.55)	1.49 (1.03–2.15)	1.48 (1.09–1.98)
Pulmonary hypertension	Yes	2.48 (1.43–4.31)	1.35 (0.89–2.05)	1.7 (1.23–2.35)
Year	2016	1	1	1
	2017	0.82 (0.36–1.86)	0.98 (0.52–1.85)	0.93 (0.57–1.53)
	2018	0.4 (0.15–1.1)	1.21 (0.67–2.18)	0.92 (0.56–1.5)
	2019	0.88 (0.39–1.99)	1.22 (0.68–2.19)	1.1 (0.69–1.76)
	2020	0.92 (0.41–2.08)	1.4 (0.77–2.56)	1.24 (0.77–1.99)
IPF	Yes	NA	NA	1.13 (0.8–1.59)

CCI: Charlson comorbidity index. OR: odds ratio. NA: not adequate.

## Data Availability

According to the contract signed with the Spanish Ministry of Health and Social Services, which provided access to the databases from the Spanish National Hospital Database (RAE-CMBD, Registro de Actividad de Atención Especializada. Conjunto Mínimo Básico de Datos, Registry of Specialized Health Care Activities. Minimum Basic Data Set), we cannot share the databases with any other investigator, and we have to destroy the databases once the investigation has concluded. Consequently, we cannot upload the databases to any public repository. However, any investigator can apply for access to the databases by filling out the questionnaire Available online: https://www.sanidad.gob.es/estadEstudios/estadisticas/estadisticas/estMinisterio/SolicitudCMBD.htm (accessed on 22 March 2023). All other relevant data are included in the paper.

## Supplementary Materials

The following supporting information can be downloaded at: https://www.mdpi.com/article/10.3390/healthcare11111534/s1, Table S1: ICD-10 codes using in this investigation; Table S2: In hospital mortality according to study variables among patients with idiopathic pulmonary fibrosis (IPF) who underwent a lung transplantation in Spain from 2016 to 2020; Table S3: Characteristics and in hospital mortality according to study variables among patients with idiopathic pulmonary fibrosis (IPF) who underwent a lung transplantation in Spain in years 2019 versus year 2020.

## References

[1] Arcasoy S.M., Kotloff R.M. (1999). Lung transplantation. N. Engl. J. Med..

[2] Kapnadak S.G., Raghu G. (2021). Lung transplantation for interstitial lung disease. Eur. Respir. Rev..

[3] Chambers D.C., Cherikh W.S., Harhay M.O., Hayes D., Hsich E., Khush K.K., Meiser B., Potena L., Rossano J.W., Toll A.E. (2019). The International Thoracic Organ Transplant Registry of the International Society for Heart and Lung Transplantation: Thirty-sixth adult lung and heart-lung transplantation Report-2019; Focus theme: Donor and recipient size match. J. Heart Lung Transplant..

[4] Chambers D.C., Zuckermann A., Cherikh W.S., Harhay M.O., Hayes D., Hsich E., Khush K.K., Potena L., Sadavarte A., Singh T.P. (2020). The International Thoracic Organ Transplant Registry of the International Society for Heart and Lung Transplantation: 37th adult lung transplantation report-2020; focus on deceased donor characteristics. J. Heart Lung Transplant..

[5] Yusen R.D., Edwards L.B., Kucheryavaya A.Y., Benden C., Dipchand A.I., Dobbels F., Goldfarb S.B., Levvey B.J., Lund L.H., Meiser B. (2014). The registry of the International Society for Heart and Lung Transplantation: Thirty-first adult lung and heart-lung transplant report-2014; focus theme: Retransplantation. J. Heart Lung Transplant..

[6] Chambers D.C., Cherikh W.S., Goldfarb S.B., Hayes D., Kucheryavaya A.Y., Toll A.E., Khush K.K., Levvey B.J., Meiser B., Rossano J.W. (2018). The International Thoracic Organ Transplant Registry of the International Society for Heart and Lung Transplantation: Thirty-fifth adult lung and heart-lung transplant report-2018; Focus theme: Multiorgan Transplantation. J. Heart Lung Transplant..

[7] Valapour M., Lehr C.J., Skeans M.A., Smith J.M., Uccellini K., Goff R., Foutz J., Israni A.K., Snyder J.J., Kasiske B.L. (2020). OPTN/SRTR 2018 Annual Data Report: Lung. Am. J. Transplant..

[8] Glass D.S., Grossfeld D., Renna H.A., Agarwala P., Spiegler P., DeLeon J., Reiss A.B. (2022). Idiopathic pulmonary fibrosis: Current and future treatment. Clin. Respir. J..

[9] Raghu G., Remy-Jardin M., Richeldi L., Thomson C.C., Inoue Y., Johkoh T., Kreuter M., Lynch D.A., Maher T.M., Martinez F.J. (2022). Idiopathic Pulmonary Fibrosis (an Update) and Progressive Pulmonary Fibrosis in Adults: An Official ATS/ERS/JRS/ALAT Clinical Practice Guideline. Am. J. Respir. Crit. Care Med..

[10] Somogyi V., Chaudhuri N., Torrisi S.E., Kahn N., Müller V., Kreuter M. (2019). The therapy of idiopathic pulmonary fibrosis: What is next?. Eur. Respir. Rev..

[11] Hackman K.L., Bailey M.J., Snell G.I., Bach L.A. (2014). Diabetes is a major risk factor for mortality after lung transplantation. Am. J. Transplant..

[12] Patti M.G., Vela M.F., Odell D.D., Richter J.E., Fisichella P.M., Vaezi M.F. (2016). The intersection of GERD, aspiration, and lung transplantation. J. Laparoendosc. Adv. Surg. Tech. A.

[13] George P.M., Patterson C.M., Reed A.K., Thillai M. (2019). Lung transplantation for idiopathic pulmonary fibrosis. Lancet Respir. Med..

[14] Balestro E., Cocconcelli E., Tinè M., Biondini D., Faccioli E., Saetta M., Rea F. (2019). Idiopathic Pulmonary Fibrosis and Lung Transplantation: When it is Feasible. Medicina.

[15] Le Pavec J., Dauriat G., Gazengel P., Dolidon S., Hanna A., Feuillet S., Pradere P., Crutu A., Florea V., Boulate D. (2020). Lung transplantation for idiopathic pulmonary fibrosis. Presse Med..

[16] Force S.D., Kilgo P., Neujahr D.C., Pelaez A., Pickens A., Fernandez F.G., Miller D.L., Lawrence C. (2011). Bilateral lung transplantation offers better long-term survival, compared with single-lung transplantation, for younger patients with idiopathic pulmonary fibrosis. Ann. Thorac. Surg..

[17] Villavicencio M.A., Axtell A.L., Osho A., Astor T., Roy N., Melnitchouk S., D’Alessandro D., Tolis G., Raz Y., Neuringer I. (2018). Single- versus Double-Lung Transplantation in Pulmonary Fibrosis: Impact of Age and Pulmonary Hypertension. Ann. Thorac. Surg..

[18] Ministerio de Sanidad, Servicios Sociales e Igualdad (2015). Real Decreto 69/2015, de 6 de Febrero, por el que se Regula el Registro de Actividad de Atención Sanitaria Especializada. (Spanish National Hospital Discharge Database). BOE.

[19] Sundararajan V., Henderson T., Perry C., Muggivan A., Quan H., Ghali W.A. (2004). New ICD-10 version of the Charlson comorbidity index predicted in-hospital mortality. J. Clin. Epidemiol..

[20] Ministerio de Sanidad, Consumo y Bienestar Social Solicitud de Extracción de datos–Extraction Request (Spanish National Hospital Discharge Database). https://www.mscbs.gob.es/estadEstudios/estadisticas/estadisticas/estMinisterio/SolicitudCMBDdocs/2018_Formulario_Peticion_Datos_RAE_CMBD.pdf.

[21] Hardman G., Sutcliffe R., Hogg R., Mumford L., Grocott L., Mead-Regan S.J., Nuttall J., Dunn S., Seeley P., Clark S. (2021). The impact of the SARS-CoV-2 pandemic and COVID-19 on lung transplantation in the UK: Lessons learned from the first wave. Clin. Transplant..

[22] Michel S., Witt C., Gottlieb J., Aigner C. (2021). Impact of COVID-19 on Lung Transplant Activity in Germany—A Cross-Sectional Survey. Thorac. Cardiovasc. Surg..

[23] Picard C., Le Pavec J., Tissot A. (2020). Impact of the COVID-19 pandemic and lung transplantation program in France. Respir. Med. Res..

[24] King T.E., Bradford W.Z., Castro-Bernardini S., Fagan E.A., Glaspole I., Glassberg M.K., Gorina E., Hopkins P.M., Kardatzke D., Lancaster L. (2014). A phase 3 trial of pirfenidone in patients with idiopathic pulmonary fibrosis. N. Engl. J. Med..

[25] Richeldi L., du Bois R.M., Raghu G., Azuma A., Brown K.K., Costabel U., Cottin V., Flaherty K.R., Hansell D.M., Inoue Y. (2014). Efficacy and safety of nintedanib in idiopathic pulmonary fibrosis. N. Engl. J. Med..

[26] Laporta Hernandez R., Aguilar Perez M., Lázaro Carrasco M.T., Ussetti Gil P. (2018). Lung Transplantation in IdiopathicPulmonary Fibrosis. Med. Sci..

[27] Wei D., Gao F., Wu B., Zhou M., Zhang J., Yang H., Liu D., Fan L., Chen J. (2019). Single versus bilateral lung transplantation for idiopathic pulmonary fibrosis. Clin. Respir. J..

[28] Grossman R.F., Frost A., Zamel N., Patterson G.A., Cooper J.D., Myron P.R., Dear C.L., Maurer J. (1990). Results of single-lung transplantation for bilateral pulmonary fibrosis. The Toronto Lung Transplant Group. N. Engl. J. Med..

[29] Thabut G., Christie J.D., Ravaud P., Castier Y., Dauriat G., Jebrak G., Fournier M., Lesèche G., Porcher R., Mal H. (2009). Survival after bilateral versus single-lung transplantation for idiopathic pulmonary fibrosis. Ann. Intern. Med..

[30] Schaffer J.M., Singh S.K., Reitz B.A., Zamanian R.T., Mallidi H.R. (2015). Single- vs double-lung transplantation in patients with chronic obstructive pulmonary disease and idiopathic pulmonary fibrosis since the implementation of lung allocation based on medical need. JAMA.

[31] Amor M., Rosengarten D., Shintenberg D., Pertzov B., Shostak Y., Kramer M. (2020). Lung Transplantation in Idiopathic Pulmonary Fibrosis: Risk Factors and Outcome. Isr. Med. Assoc. J..

[32] Li D., Liu Y., Wang B. (2020). Single versus bilateral lung transplantation in idiopathic pulmonary fibrosis: A systematic review and meta-analysis. PLoS ONE.

[33] Spratt J.R., Tomic R., Brown R.Z., Rudser K., Loor G., Hertz M., Shumway S., Kelly R.F. (2019). Single versus Bilateral Lung Transplantation for Idiopathic Pulmonary Fibrosis in the Lung Allocation Score Era. J. Surg. Res..

[34] Adegunsoye A., Strek M., Garrity E., Guzy R., Bag R. (2017). Comprehensive Care of the Lung Transplant Patient. CHEST.

[35] Balestro E., Calabrese F., Turato G., Lunardi F., Bazzan E., Marulli G., Biondini D., Rossi E., Sanduzzi A., Rea F. (2016). Immune Inflammation and Disease Progression in Idiopathic Pulmonary Fibrosis. PLoS ONE.

[36] Bosa S., Vosa R., Van Raemdonck D., Verledena G. (2020). Survival in adult lung transplantation: Where are we in 2020?. Curr. Opin. Organ. Transplant..

[37] Brouckaert J., Verleden S.E., Verbelen T., Coosemans W., Decaluwé H., De Leyn P., Depypere L., Nafteux P., Van Veer H., Meyns B. (2019). Double-lung versus heart-lung transplantation for precapillary pulmonary arterial hypertension: A 24-year single-center retrospective study. Transpl. Int..

[38] Diamond J.M., Lee J.C., Kawut S.M., Shah R.J., Localio A.R., Bellamy S.L., Lederer D.J., Cantu E., Kohl B.A., Lama V.N. (2013). Clinical risk factors for primary graft dysfunction after lung transplantation. Am. J. Respir. Crit. Care Med..

[39] Hoetzenecker K., Benazzo A., Stork T., Sinn K., Schwarz S., Schweiger T., Klepetko W., Vienna Lung Transplant Group (2020). Bilateral lung transplantation on intraoperative extracorporeal membrane oxygenator: An observational study. J. Thorac. Cardiovasc. Surg..

[40] Snell G.I., Yusen R.D., Weill D., Strueber M., Garrity E., Reed A., Pelaez A., Whelan T.P., Perch M., Bag R. (2017). Report of the ISHLT Working Group on Primary Lung Graft Dysfunction, part I: Definition and grading—A 2016 Consensus Group statement of the International Society for Heart and Lung Transplantation. J. Heart Lung Transplant..

[41] Loor G., Mattar A., Schaheen L., Bremner R. (2022). Surgical Complications of Lung Transplantation. Thorac. Surg. Clin..

[42] Aguilar-Guisado M., Givaldá J., Ussetti P., Ramos A., Morales P., Blanes M., Bou G., de la Torre-Cisneros J., Román A., Borro J.M. (2007). Pneumonia after lung transplantation in the RESITRA Cohort: A multicenter prospective study. Am. J. Transplant..

[43] Zhou J., Huang H., Liu S., Yu P., Wan Q. (2015). *Staphylococcus aureus* bacteremias following liver transplantation: A clinical analysis of 20 cases. Ther. Clin. Risk Manag..

[44] Yun J.H., Lee S.O., Jo K.W., Choi S.H., Lee J., Chae E.J., Do K.H., Choi D.K., Choi I.C., Hong S.B. (2015). Infections after lung transplantation: Time of occurrence, sites, and microbiologic etiologies. Korean J. Intern. Med..

[45] Joean O., Welte T., Gottlieb J. (2022). Chest Infections after Lung Transplantation. Chest.

[46] Bunsow E., Los-Arcos I., Martin-Gómez M.T., Bello I., Pont T., Berastegui C., Ferrer R., Nuvials X., Deu M., Peghin M. (2020). Donor-derived bacterial infections in lung transplant recipients in the era of multidrug resistance. J. Infect..

[47] Chang A., Musk M., Lavender M., Wrobel J., Yaw M.C., Lawrence S., Chirayath S., Boan P. (2019). Cytomegalovirus viremia in lung transplantation during and after prophylaxis. Transpl. Infect. Dis..

[48] Prudencio-Ribera V.C., Corral-Blanco M., Jarrín-Estupiñán M.E., Alonso-Moralejo R., Pérez-González V., Martínez-Serna I., González-Serrano M., De Pablo-Gafas A. (2019). Analysis of Intrahospital Mortality in Patients with Lung Transplant Due to Diffuse Parenchymal Lung Disease. Transplant. Proc..

[49] Arango Tomás E.A., Algar Algar F.J., Cerezo Madueno F., Salvatierra Valazquez A. (2015). Evolution and risk factors for early mortality after lung transplantation for idiopathic pulmonary fibrosis: An experience of 20 years. Transplant. Proc..

[50] Chambers D.C., Perch M., Zuckermann A., Cherikh W.S., Harhay M.O., Hayes D., Hsich E., Khush K.K., Potena L., Sadavarte A. (2021). The International Thoracic Organ Transplant Registry of the International Society for Heart and Lung Transplantation: Thirty-eighth adult lung transplantation report-2021; Focus on recipient characteristics. J. Heart Lung Transplant..

[51] Urlik M., Stącel T., Latos M., Antończyk R., Ferens M., Zawadzki F., Król B., Pasek P., Przybyłowski P., Zembala M. (2020). Donor-related Risk Factors Associated with Increased Mortality after Lung Transplant. Transplant. Proc..

[52] Mosher C., Weber J., Frankel C., Neely M., Palmer S. (2021). Risk factors for mortality in lung transplant recipients aged ≥65 years: A retrospective cohort study of 5,815 patients in the scientific registry of transplant recipients. J. Heart Lung Transplant..

[53] Lund L.H., Edwards L.B., Kucheryavaya A.Y., Benden C., Dipchand A.I., Goldfarb S., Levvey B.J., Meiser B., Rossano J.W., Yusen R.D. (2015). The Registry of the International Society for Heart and Lung Transplantation: Thirty-second Official Adult Heart Transplantation Report—2015; Focus Theme: Early Graft Failure. J. Heart Lung Transplant..

[54] Fielding-Singh V., Grogan T.R., Neelankavil J.P. (2021). Accuracy of administrative database estimates of national surgical volume: Solid organ transplantation in the National Inpatient Sample. Clin. Transplant..

[55] Registro Español de Trasplante Pulmonar Resultados 2001–2020. http://www.ont.es/infesp/Documents/MEMORIA%20ANUAL%20RETP%202001-2020.pdf.

[56] Merlo C.A., Weiss E.S., Orens J.B., Borja M.C., Diener-West M., Conte J.V., Shah A.S. (2009). Impact of U.S. Lung Allocation Score on survival after lung transplantation. J. Heart Lung Transplant..

