# Supplementary material for: Trends and Outcomes in Lung Transplantation in Patients with and without Idiopathic Pulmonary Fibrosis in Spain during the Period 2016–2020
Source: Healthcare (Basel). 2023 May 24;11(11):1534. doi: 10.3390/healthcare11111534 (PMC10252271; doi:10.3390/healthcare11111534)
Supplement: Supplementary file 1 [file healthcare-11-01534-s001.zip › healthcare-2373815-supplementary.pdf]

Table S1. ICD-10 codes using in this investigation.

DIAGNOSIS	ICD-10 Codes
Single lung transplantation	0BYCxxx, 0BYDxxx, 0BYFxxx, 0BYGxxx, 0BYHxxx, 0BYJxxx, 0BYKxxx 0BYLxxx
Bilateral lung transplantation	0BYMxxx
COVID-19	B34.2, B97.29, U07.1
Pulmonary hypertension	I27.0, I27.2x, I27.89
Haemodialysis	5A1Dxxx
Extracorporeal membrane oxygenation	5A15xxx
Tracheostomy	0B11xxx
Lung transplant rejection	T86.810
Lung transplant failure	T86.811
Lung transplant infection	T86.812
Other or unspecified complications of lung transplant	T86.818, T86.819
Pneumonia	J13-J18
Asthma	J45
Current tobacco use	Z72.0, F17.21x
Ventilator-associated pneumonia	J95.851
Staphylococcus bacteraemia	A4101, A4102, A411, A412, A4901, A4902, B9561, B9562, B957, B958
Gram-negative bacteraemia	A413, A4150, A4151, A4152, A4153, A4159, B961, B9620, B9621, B9622, B9623, B9629, B963, B964, B965
Pseudomonas aeruginosa infection	B96.5
Aspergillus infection	B44.xx
Cytomegalovirus infection	B25.x

Table S2. In hospital mortality according to study variables among patients with idiopathic pulmonary fibrosis (IPF) who underwent a lung transplantation in Spain from 2016 to 2020.

		2016	2017	2018	2019	2020	TOTAL
Type, n (%)	Single	8(11.76)	12(14.12)	4(7.84)	5(9.62)	8(22.86)	37(12.71)
	Bilateral	5(17.86)	3(8.11)	3(6)	11(13.58)	9(10.47)	31(10.99)
Sex, n(%)	Men	6(8.45)	9(10)	5(6.67)	12(12.5)	15(15)	47(10.88)
	Women	7(28)	6(18.75)	2(7.69)	4(10.81)	2(9.52)	21(14.89)
Age groups, n(%)	<45 years	2(18.18)	2(18.18)	1(16.67)	1(8.33)	1(20)	7(15.56)
	45-54 years	3(21.43)	4(20)	1(6.25)	1(4.76)	4(23.53)	13(14.77)
	55-64 years	8(15.38)	8(12.7)	3(5)	12(16.67)	8(11.11)	39(12.23)
	≥ 65 years	0(0)	1(3.57)	2(10.53)	2(7.14)	4(14.81)	9(7.44)
CCI, n(%)	0	7(13.21)	5(9.09)	4(8.33)	6(9.84)	4(7.02)	26(9.49)
	≥1	6(13.95)	10(14.93)	3(5.66)	10(13.89)	13(20.31)	42(14.05)
Pulmonary hypertension, n(%)[a]	Yes	3(14.29)	5(17.24)	4(16)	6(31.58)	10(25)	28(20.9)
	No	10(13.33)	10(10.75)	3(3.95)	10(8.77)	7(8.64)	40(9.11)
Any complication of lung transplant, n(%)	Yes	7(19.44)	10(17.24)	4(6.25)	8(11.27)	11(17.46)	40(13.7)
	No	6(10)	5(7.81)	3(8.11)	8(12.9)	6(10.34)	28(9.96)

CCI: Charlson comorbidity index.

[a]p value <0.05 when comparing total values between categories.

Table S3. Characteristics and in hospital mortality according to study variables among patients with idiopathic pulmonary fibrosis (IPF) who underwent a lung transplantation in Spain in years 2019 versus year 2020.

		**Characteristics**			**IHM**		
		**2019**	**2020**	**P value**	**2019**	**2020**	**P value**
Number of procedures		133	121	0.403	16(12.03)	17(14.05)	0.633
Type, n (%)	Single	52(39.1)	35(28.93)	0.088	5(9.62)	8(22.86)	0.089
	Bilateral	81(60.9)	86(71.07)		11(13.58)	9(10.47)	0.535
Sex, n(%)	Men	96(72.18)	100(82.64)	0.047	12(12.5)	15(15)	0.612
	Women	37(27.82)	21(17.36)		4(10.81)	2(9.52)	0.877
Age, mean (SD)		58.21(9.24)	59.06(8.95)	0.459	59.56(6.81)	59.12(7.72)	0.862
Age groups, n(%)	<45 years	12(9.02)	5(4.13)	0.430	1(8.33)	1(20)	0.496
	45-54 years	21(15.79)	17(14.05)		1(4.76)	4(23.53)	0.089
	55-64 years	72(54.14)	72(59.5)		12(16.67)	8(11.11)	0.335
	≥ 65 years	28(21.05)	27(22.31)		2(7.14)	4(14.81)	0.362
CCI, mean (SD)		0.81(0.96)	0.86(1.17)	0.756	1(1.1)	1.24(1.09)	0.541
CCI, n(%)	0	61(45.86)	57(47.11)	0.843	6(9.84)	4(7.02)	0.583
	≥1	72(54.14)	64(52.89)		10(13.89)	13(20.31)	0.319
Pulmonary hypertension n(%)	Yes	19(14.29)	40(33.06)	<0.001	6(31.58)	10(25)	0.595
	No	114(85.71)	81(66.94)		10(8.77)	7(8.64)	0.975
Any complication of lung transplant, n(%)	Yes	71(53.38)	63(52.07)	0.834	8(11.27)	11(17.46)	0.305
	No	62(46.62)	58(47.93)		8(12.9)	6(10.34)	0.663

CCI: Charlson comorbidity index.
